# Identification of pOENI-1 and Related Plasmids in *Oenococcus oeni* Strains Performing the Malolactic Fermentation in Wine

**DOI:** 10.1371/journal.pone.0049082

**Published:** 2012-11-05

**Authors:** Marion Favier, Eric Bilhère, Aline Lonvaud-Funel, Virginie Moine, Patrick M. Lucas

**Affiliations:** 1 University of Bordeaux, ISVV, Unit of Enology EA 4577, Villenave d'Ornon, France; 2 SARCO, research subsidiary of the Laffort group, BP 40, Bordeaux, France; 3 Laffort, BP 17, Bordeaux, France; The University of Hong Kong, Hong Kong

## Abstract

Plasmids in lactic acid bacteria occasionally confer adaptive advantages improving the growth and behaviour of their host cells. They are often associated to starter cultures used in the food industry and could be a signature of their superiority. *Oenococcus oeni* is the main lactic acid bacteria species encountered in wine. It performs the malolactic fermentation that occurs in most wines after alcoholic fermentation and contributes to their quality and stability. Industrial *O. oeni* starters may be used to better control malolactic fermentation. Starters are selected empirically by virtue of their fermentation kinetics and capacity to survive in wine. This study was initiated with the aim to determine whether *O. oeni* contains plasmids of technological interest. Screening of 11 starters and 33 laboratory strains revealed two closely related plasmids, named pOENI-1 (18.3-kb) and pOENI-1v2 (21.9-kb). Sequence analyses indicate that they use the theta mode of replication, carry genes of maintenance and replication and two genes possibly involved in wine adaptation encoding a predicted sulphite exporter (*tauE*) and a NADH:flavin oxidoreductase of the old yellow enzyme family (*oye*). Interestingly, pOENI-1 and pOENI-1v2 were detected only in four strains, but this included three industrial starters. PCR screenings also revealed that *tauE* is present in six of the 11 starters, being probably inserted in the chromosome of some strains. Microvinification assays performed using strains with and without plasmids did not disclose significant differences of survival in wine or fermentation kinetics. However, analyses of 95 wines at different phases of winemaking showed that strains carrying the plasmids or the genes *tauE* and *oye* were predominant during spontaneous malolactic fermentation. Taken together, the results revealed a family of related plasmids associated with industrial starters and indigenous strains performing spontaneous malolactic fermentation that possibly contribute to the technological performance of strains in wine.

## Introduction

Lactic acid bacteria (LAB) contribute to winemaking during the malolactic fermentation (MLF). MLF usually takes place after the yeast-driven alcoholic fermentation (AF) and lasts a few days to several months depending on wine composition, temperature and LAB population [1], [2]. MLF mainly consists in the conversion of the strong dicarboxylic L-malate into the softer L-lactate and CO_2_. It is beneficial in that it reduces the acidity of wine, improves its taste and aromas and contributes to its microbiological stability [3], [4]. MLF generally occurs when the LAB population exceeds 10^6^ cells.ml^−1^. *Oenococcus oeni* is often the only species detected in wine during MLF and thus considered as the best-adapted species [5]. At the intraspecies level, *O. oeni* strains differ considerably in terms of capacity to survive in wine and to conduct MLF. They are more or less tolerant to wine acidity (pH 2.9–4.0), alcohol (11–17%), phenols and sulfites [6], [7]. They also perform MLF more or less rapidly and have a beneficial or detrimental impact on wine aromas [8], [9]. To better control the onset, duration and aromatic impact of MLF, winemakers can make use of industrial *O. oeni* strains. A few dozens of malolactic starters are available to date. They are natural strains selected on the basis of their tolerance to wine stressors, kinetics of MLF, aromas production and safety regarding undesirable metabolisms such as the production of biogenic amines, bitterness or ropiness [10].

The molecular mechanisms at the origin of *O. oeni* survival and growth in wine, and differences existing between strains are still poorly understood. Diverse genes possibly involved in wine adaptation were described in the past decades. This includes genes related to general stress response, membrane composition and fluidity, pH homeostasis, multidrug resistance, or response to oxidative stress and DNA damage [11]–[18]. Comparative genomic analyses also revealed a number of genes that were statistically more often present in *O. oeni* strains of technological interest [19], [20], [21]. However, all the genes identified to date do not satisfactorily explain differences of survival in wine and kinetics of MLF observed amongst *O. oeni* strains [22].

Until now, little attention was paid to the plasmids of *O. oeni*. Plasmids are known as a source of phenotypic and genetic diversity in LAB and occasionally confer adaptive advantages to host strains. Besides maintenance and transfer mechanisms, they encode important traits such as secondary metabolisms, resistance to bacteriophages, antibiotics or heavy metals, and production of exopolysaccharides, bacteriocins and immunity proteins [23], [24]. Plasmids are often associated to starter cultures used in the food industry and could be a signature of their technological superiority and individuality [24]. In the dairy starter *Lactococcus lactis*, they confer phenotypes that reflect adaptation to the dairy environment, such as lactose catabolism, protease activity, peptide and amino acid uptake and bacteriophage resistance [25]. Six small cryptic plasmids of *O. oeni* were sequenced and described to date: pLo13 [26], p4028 [27], pOg32 [28], pRS1 [29], pRS2 and pRS3 [30]. They encode replication and mobilization proteins but do not carry any gene potentially involved in wine adaptation. Large plasmids were detected in a number of *O. oeni* strains but no sequence was reported to date [31]–[35]. A functional role has been assigned to the 22.5-kb plasmid pBL34 that seems to confer pesticide resistance to its *O. oeni* host cells [35].

This work was initiated with the aim to investigate whether plasmids may contribute to wine adaptation of *O. oeni* strains. Two large plasmids, named pOENI-1 (18.3 kb) and pOENI-1v2 (21.9 kb), were described for the first time in *O. oeni*. Their contribution to the technological properties of strains was investigated by analyzing their sequences, their distribution in the *O. oeni* species, their associated phenotypes and their frequency in wines at different steps of winemaking.

## Materials and Methods

### Bacteria strains and culture conditions

*O. oeni* strains used in this study are listed in Table 1. They consist in 11 industrial starters, 14 strains of laboratory collections and 18 strains isolated for this study from red and white wines collected during spontaneous MLF of vintages 2008 and 2009. New isolates were deposited in the SARCO collection (SARCO Laboratory, Bordeaux, France). All the strains were stored at −80°C in the presence of 30% (v/v) glycerol and propagated under anaerobic conditions at 25°C in grape juice medium (GJ) containing 25% (v/v) commercial red grape juice, 0.5% (wt/v) yeast extract, 0.1% (v/v) Tween 80, pH 4.8.

**Table 1:** *O. oeni* strains used in this study.

Strain^a^	Collection	Origin
**C1**	IOEB	Commercial product Lactooenos 350 Preac, Laffort
**C2**	IOEB	Commercial product Lactooenos 450 Preac, Laffort
**C3**	IOEB	Commercial product Lactooenos B16, Laffort
**C4**	IOEB	Commercial product Vitilactic BL01, Martin Vialatte
**C5**	IOEB	Commercial product Viniflora Ciné, CHR Hansen
**C6**	IOEB	Commercial product Lalvin 31, Lallemand
**C7**	IOEB	Commercial product Oeno2, Lamothe-Abiet
**C8**	IOEB	Commercial product Lactoenos SB3, Laffort
**C9**	IOEB	Commercial product Vitilactic F, Matin Vialatte
**C10**	IOEB	Commercial product Lalvin VP41, Lallemand
**PSU1**	ATCC	Commercial starter, Red wine, California, 1977
**IOEB 0026**	IOEB	Red wine, France, 2000
**IOEB 0501**	IOEB	Red wine, France, 2005
**IOEB 0608**	IOEB	Red wine, France, 2006
**IOEB 8419**	IOEB	Red wine, France, 1984
**IOEB 9115**	IOEB	Red wine, France, 1991
**IOEB 9304**	IOEB	Cider, France, 1993
**IOEB 89006**	IOEB	Red wine, France, 1989
**IOEB 89127**	IOEB	Red wine, France, 1989
**IOEB S268**	IOEB-SARCO	Red wine, France, 2000
**IOEB S384**	IOEB-SARCO	White wine, France, 2002
**IOEB S422**	IOEB-SARCO	White wine, France, 2002
**IOEB S343a**	IOEB-SARCO	Red wine, France, 2002
**IOEB S455**	IOEB-SARCO	White wine, France, 2003
**S4**	SARCO	Red wine, France, 2009
**S11**	SARCO	Sparkling white wine, France, 2008
**S12**	SARCO	White wine, France, 2009
**S13**	SARCO	Red wine, France, 2009
**S14**	SARCO	Red wine, France, 2009
**S15**	SARCO	Red wine, France, 2009
**S17**	SARCO	Red wine, France, 2009
**S18**	SARCO	Red wine, France, 2009
**S19**	SARCO	Red wine, France, 2009
**S20**	SARCO	Red wine, France, 2009
**S22**	SARCO	White wine, France, 2009
**S23**	SARCO	White wine, England, 2009
**S24**	SARCO	Red wine, England, 2009
**S25**	SARCO	Red wine, France, 2009
**S26**	SARCO	Red wine, France, 2009
**S27**	SARCO	Red wine, France, 2009
**S28**	SARCO	Red wine, France, 2009
**S29**	SARCO	Red wine, France, 2009
**ATCC BAA 1163**	ATCC	Red wine, France, 1984

a IOEB: Institute of oenology of Bordeaux, S: SARCO, ATCC: American type culture collection.

### Strain typing

Strain typing was performed by NotI restriction of bacterial DNA followed by pulse field gel electrophoresis (PFGE) of restriction fragments as previously described [36]. DNA restriction patterns were compared in a dendrogram generated by the unweighted pair group method using arithmetic means (UPGMA) with the Dice coefficient of similarity and a tolerance limit of 2.3% in Bionumerics 5.1 software (Applied Maths, Kortrijk, Belgium). Multilocus sequence typing (MLST) was also performed for several strains according to the procedure described in [36]. MLST data was processed in a neighbor-joining tree constructed using MEGA4 [37].

### Plasmid sequencing and analysis

Plasmid pOENI-1 was isolated from a 10-ml culture of commercial strain *O. oeni* C9 by the alkaline lysis method [38]. The plasmid DNA preparation was digested by EcoRI and PstI (New Englands Biolabs) to construct a library of inserts in the *E. coli* vector pBluescript II SK+ (Stratagene). Two inserts (1.3 and 2.5 kb) were sequenced. The rest of the plasmid was amplified in two PCR products (4.0 and 10.5 kb) obtained using primers designed in the first sequences and a high-fidelity DNA polymerase (iProof, Bio-Rad). The two PCR products were cloned in pGEM-T Easy (Promega), transferred in *E. coli* and sequenced. The sequences were assembled using Lasergene (DNA Star) in a single circular DNA molecule of 18,332-bp. The sequence of plasmid pOENI-1v2 was obtained by sequencing the genome of strain S11 by the 454 technology (single reads of 450-nt on average, GenoToul, Toulouse, France). Sequences assembled with Lasergene (DNA-Star) formed a circular DNA molecule of 21,926-bp (coverage >100X all along the plasmid sequence). Open reading frames (ORFs) were predicted with GeneMark [39] and Glimmer [40]. Gene annotation was performed manually using Blast and Interproscan analyses [41].

### PCR-based detection of pOENI-1 in *O. oeni* strains

Bacterial genomic DNAs were extracted using the Wizard genomic DNA purification kit (Promega) according to the manufacturer's instructions. DNA preparations were used as template in PCR assays to detect the plasmid genes *repA*, *oye* and *tauE*, the chromosomal gene OEOE_0812 (locus tag of the *O. oeni* PSU-1 genome, NC_008528), and to confirm the integrity of plasmids using a combination of three overlapping PCRs that extend over the whole plasmid sequences. The primers used are listed in Table 2. PCR amplifications were performed in 20-µl mixtures containing 25 ng of template DNA, 0.25 µM of each primer and the Taq-&GO^TM^ PCR mix (MP Biomedicals). The standard PCR program was 95°C for 5 min; followed by 30 cycles of 95°C for 30 s, 50°C to 60°C for 30 s, 72°C for 30 s and a final step of 10 min at 72°C. PCR products were visualized under UV light exposure after electrophoresis in 1.2% (w/v) agarose gels and staining with ethidium bromide.

**Table 2:** Primers list.

Primer name	Forward sequence (5′–3′)	Reverse sequence (5′–3′)	Target	Product (bp)
***Detection of plasmid genes***				
**8a/8b** a	TAAGCAAACGGGGTCAACTC	TCAGGCCGAGGATCAATAAC	ORF 8	142
**oye1/oye2**	AGTAGTTATTCCGCCAATGA	ATGAATGGCTCCTTAGCATA	ORF 11	602
**oyeQ1/oyeQ2** a	TAAGGGATTTGAAGGCCAACT	TTGAAGAATTGCTTTAGCACCA	ORF 11	106
**repA1/repA2**	ATCGGCTCGAATATTCTCTCAA	CGTATTCTCTAGCCGCTTGTTT	ORF 15	911
**2repA/NC1**	ATCACCTAGTAGACGAAGAG	GGTAGGCAGGTTCTAATC	ORF 15_ORF 1	6464
**orf20a/orf10**	AGTTAAGAACTATCGTAAGTCC	TTACTGGCCTCCTACTGAAC	ORF 20_ORF 10	9848
**repA2/NC3**	CGTATTCTCTAGCCGCTTGTTT	GCATTCGACTTTGCGGAATG	ORF 10_ORF 15	5199/8765^b^
**oye1/orf13-2**	AGTAGTTATTCCGCCAATGA	TACAGCATACACTCACAGCA	ORF 11_ORF 13	2376/5942^b^
**orf20a/orf20b**	AGTTAAGAACTATCGTAAGTCC	AACAGGATCATAGTACATCAC	ORF20	821
***Detection of chromosomal genes***				
**OO1/OO2**	GTGCCGCTTTTTTGGATATTA	AGCAATTTTATCTTTATAGCT	*mleA*	431
**0812a/0812b**	GATTATTACCAATTCGGCTG	ACGCCGGAAATAATGTAG	OEOE_0812	540
**rpoB1/rpoB2** a	ATGGAACGTGTTGTCCGCGA	GGATTGGTTTGATCCATGAA	*rpoB*	148

a Primers used in qPCR assays.

b Product sizes obtained for pOENI-1 and pOENI-1v2.

### Determination of plasmid/*oye* gene copy number per cell

Copy number of plasmids pOENI-1, pOENI-1v2 and gene *oye* (ORF 11) per cell was determined by quantitative real-time PCR (qPCR) using a GoTaq® qPCR Master Mix (Promega) on a CFX96^TM^ Real-Time Detection System (Bio-Rad). Amplification conditions were 95°C for 3 min, followed by 40 cycles of 95°C for 30 s, 60°C for 30 s and 72°C for 30 s and a final step of 70°C to 90°C with an increment of 0.5°C each 5 s. Two primer pairs (Table 2) were used to quantify the chromosomal gene *rpoB* and the plasmid gene *oye* in order to calculate their relative proportion. Serial decimal dilutions of *O. oeni* ATCC BAA 1163 genomic DNA were used to produce the standard curves. In this strain, both *oye* and *rpoB* are present on the chromosome at one copy per cell. Standard curve equations and coefficients of correlation calculated from three independent experiments were: C_T_  = −3.33x +35.11, R^2^ = 0.996 (*rpoB*), C_T_  = −3.49x +37.28, R^2^ = 0.993 (*oye*). Genomic DNA of all tested strains was extracted from bacterial colonies suspended in 200-µl sterile H_2_O and heating for 10 min at 80°C prior cooling on ice. All determinations were done in triplicates.

### Quantification of *oye/tauE* genes in wine

Determinations were performed by qPCR as described above, except that template DNAs were extracted from total microorganisms of 10-ml samples of must or wine by the method reported in [31]. Standard curves were produced using DNA extracted from decimal dilutions of *O. oeni* ATCC BAA 1163 or S24 (*rpoB* and *tauE* at one copy per cell) inoculated in sterile wine (10^7^ to 10^2^ cells.ml^−1^). The corresponding equations indicate cycle threshold values for 1 ml of wine: ATCC BAA 1163, C_T_  = −3.61x +41.66, R^2^ = 0.906 (*rpoB*), C_T_  = −3.64x +42.26, R^2^ = 0.961 (*oye*); S24, C_T_  = −3.98x +39.38, R^2^ = 0.975 (*rpoB*), C_T_  = −3.86x +39.14, R^2^ = 0.979 (*tauE*). The tested samples were 95 red wines and musts collected at different stages of winemaking (must, alcoholic fermentation, MLF) in 86 wineries of Bordeaux's area. No industrial strain was employed to conduct MLF in these wines.

### Plasmid curing

*O. oeni* strains carrying pOENI-1 or pOENI-1v2 were cultivated in GJ medium for about 20 generations. Cultures were plated to analyze 30 colonies and determine the presence or absence of the plasmids. DNA templates were prepared by suspending each colony in 200-µl sterile H_2_O and heating for 10 min at 80°C prior cooling on ice. Multiplex PCR were performed in order to detect simultaneously a chromosomal gene (PCR positive control, *mleA*) and a plasmid gene (ORF 20). PCRs were carried out in 20-µl reaction mixtures containing 1-µl of cell suspension, 0.25 µM of each primer and the Taq-&GO^TM^ PCR mix (MP Biomedicals). Clones without plasmid were controlled by NotI-PFGE typing as described above and compared with parental strains.

### Population dynamics during growth in wine and MLF kinetics

*O. oeni* strains carrying pOENI-1 or pOENI-1v2 and isogenic plasmid-less derivatives were used in two types of experiments: direct inoculation in wine for monitoring MLF kinetics and inoculation in grape juice for monitoring cell growth in wine and MLF kinetics. For the first experiment, cells were produced as freeze-dried industrial preparations (SARCO) and inoculated to 10^7^cell.ml^−1^ in a red wine immediately after alcoholic fermentation. Wines were incubated at 20°C until completion of MLF. L-malic acid degradation was determined twice per week using an L-malic enzymatic kit (Roche Boehringer, R-biopharm). LAB populations were determined by plating on GJ medium once per week. For the second experiment, cells were propagated in GJ medium and inoculated to 10^3^ cell.ml^−1^ in a commercial grape juice supplemented with glucose/fructose to 210 g.l^−1^, L-malic acid to 4 g.l^−1^, SO_2_ to 20 mg.l^−1^ and adjusted to pH 3.6. Alcoholic fermentation was initiated by inoculating the industrial starter yeast F33 to 200 mg.l^−1^ according to manufacturer's instructions (Laffort, France). AF was monitored daily by weight-loss determinations. MLF and bacterial populations were determined three times per week as described above. Plasmids stability was investigated by PCR-analysis (see the paragraph “plasmid curing”) of 30 to 50 colonies picked up on plates produced at the inoculation time, at the end of AF and the end of MLF.

### Sequence accession numbers

The nucleotide sequences of pOENI-1 and pOENI-1v2 were submitted to GenBank and are available under the accession numbers JX416328 and JX416329, respectively.

## Results

### Sequence analysis of pOENI-1

**Figure 1:**
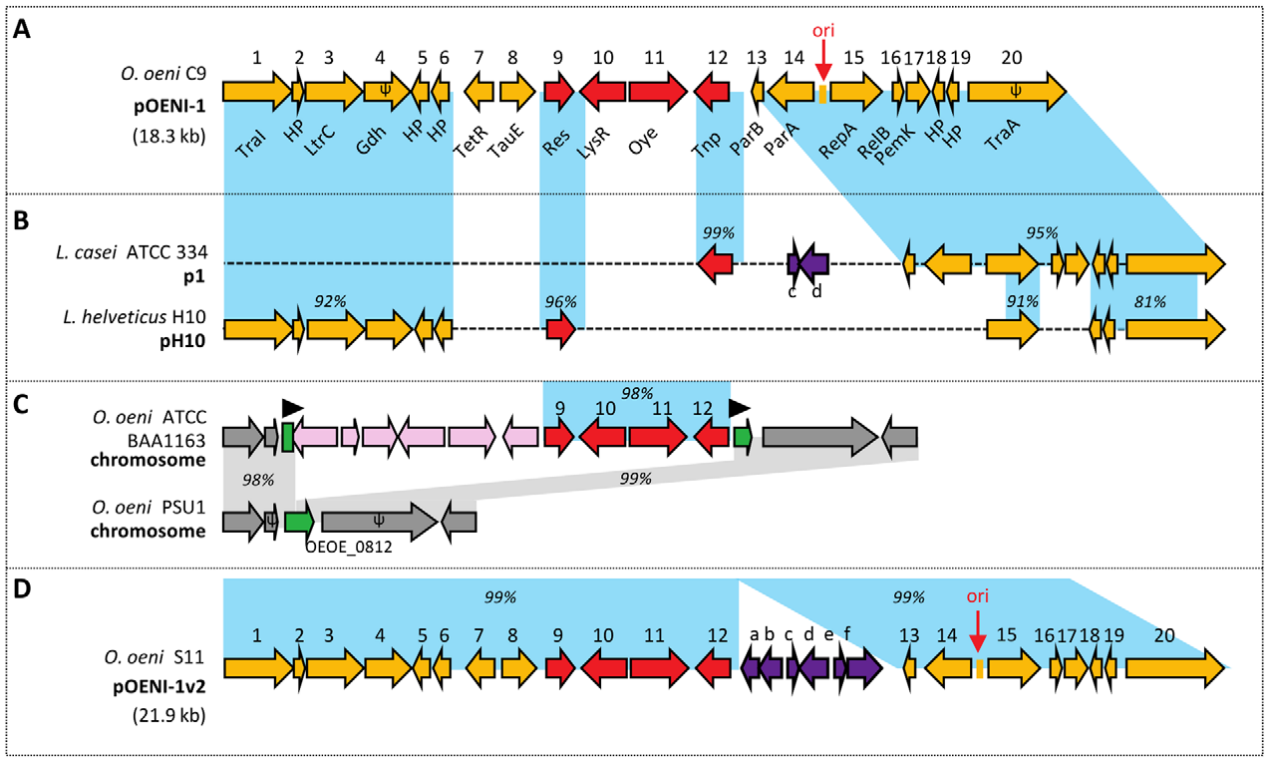
Genetic organization of pOENI-1 and comparison with related sequences. A. Genetic organization of plasmid pOENI-1. ORFs are represented by numbered arrows and identified by corresponding protein tags (see also Table 3). B. Sequence comparison of pOENI-1 and related plasmids p1 (CP000424) and pH10 (CP002430). ORFs “c, d” (purple arrows) share 99% similarity with ORFs of pOENI-1v2. C. Portions of chromosomes in *O. oeni* ATCC BAA 1163 and *O. oeni* PSU1. The gene OEOE_0812 in *O. oeni* PSU1 (green arrow) is disrupted in *O. oeni* ATCC BAA 1163 by an 10 genes insert comprising four genes conserved in pOENI-1 (red arrows) and six genes unrelated to pOENI-1 (pink arrows). The insert is bordered by an 8-bp repeated sequence (dark triangles). D. Genetic organization of pOENI-1v2. ORFs numbered from 1 to 20 share more than 99% nucleotide sequence similarity with corresponding ORFs in pOENI-1. ORFs shaded in purple are not detected in pOENI-1 and code for transposases (a, e, f,), hypothetical proteins (b, c) and a recombinase (d). Pseudogenes are symbolized by arrowheads containing the symbol ψ. Regions of sequence similarity are indicated in percentages and shaded in blue. ori: putative origin of replication.

During a survey of *O. oeni* strains, we have detected a large plasmid in the industrial strain *O. oeni* C9. This plasmid, named pOENI-1, was analyzed to determine whether it contributes to the technological properties of *O. oeni* C9. Its complete sequence was obtained by sequencing diverse restriction fragments and PCR products. pOENI-1 is a circular DNA molecule of 18,332-bp in length. Its GC% is 40.8, compared to 38% in the *O. oeni* chromosome [19]. Sequence annotation revealed 18 complete ORFs and two truncated ORFs (ORFs 4 and 20) ranging from 210 to 1512-bp (Fig. 1A and Table 3). A function was ascribed to 15 of the 20 encoded proteins. The protein encoded by ORF 15 shares more than 70% sequence identity with replication initiator protein A (RepA) encountered in theta type plasmids plca36 of *Lactobacillus casei* Zhang [42], pLgLA39 of *Lactobacillus gasseri* LA39 [43] and pSF118-44 of *Lactobacillus salivarius* UCC118 [44]. The intergenic region located between ORF 14 and ORF 15 shows all the hallmarks of the theta-type replication origin [45], [46]. It is located upstream of *repA*, contains an AT-rich region (positions 12,832–12,915, 71% AT) and an 18-bp repetition present at 7 copies (atatatctgatatatcaa, positions 12,862–12,987). Therefore, it is likely that pOENI-1 is the first large theta-type plasmid described in *O. oeni*.

**Table 3:** ORFs and predicted proteins of pOENI-1.

ORF	Position	%GC	Protein	Size (aa)	Predicted function	Best blast (organism, GenBank accession)	% identity
**1**	108-1619	44,0	TraI	503	DNA topoisomerase IA, TraI	*L. pentosus*, CCB84017	95
**2**	1742-1957	38,9	HP	71	Hypothetical protein	*L. brevis*, ZP_03940833	90
**3**	1961-3082	42,7	LtrC	373	LtrC-like protein	*L. helveticus*, ADX71206	96
**4** a	3096-4053	36,9	Gdh	319	Glycerate dehydrogenase	*Lc. kimchii*, YP_003621246	70
**5**	4558-4172	42,1	HP	128	Hypothetical protein	*P. claussenii*, AEV96201	94
**6**	4871-4551	40,5	HP	106	Hypothetical protein	*L. brevis*, ZP_03940921	99
**7**	5865-5281	52,7	TetR	194	Transcriptional regulator, TetR	*L.mali*, ZP_09449471	99
**8**	6041-6787	53,3	TauE	248	Putative permease TauE	*L. mali*, ZP_09449472	99
**9**	7365-7952	41,8	Res	195	Resolvase	*L. crispatus*, YP_003601011	99
**10**	9124-8192	35,3	LysR	310	Transcriptional regulator, LysR	*O. oeni*, ZP_01543901	100
**11**	9267-10448	42,1	Oye	393	NADH: flavin oxidoreductase	*O. oeni*, ZP_01543900	100
**12**	11258-10575	45,5	Tnp	227	Transposase	*O. oeni*, ZP_01543898	100
**13**	11927-11649	41,6	ParB	92	Partition protein, ParB	*L. casei*, YP_794449	100
**14**	12831-11908	40,8	ParA	307	Partition protein, ParA	*L. casei*, YP_794448	99
**15**	13334-14449	39,5	RepA	371	Replication protein, RepA	*P. claussenii*, AEV96162	76
**16**	14709-14990	42,9	RelB	93	Addiction module antitoxin, RelB	*L. hilgardii*, ZP_03954201	100
**17**	14980-15486	35,9	PemK	168	Addiction module toxin, PemK	*L. paracasei*, ABA12818	99
**18**	15754-15476	34,8	HP	92	Hypothetical protein	*L. brevis*, YP_796419	97
**19**	15985-15776	30,0	HP	69	Hypothetical protein	*L. mali*, ZP_0944951	90
**20** a	16256-18317	38,7	TraA	687	Putative nickase, TraA	*L. pentosus*, CCC15328	95

a pseudogenes, the characteristics of hypothetical full-length genes and proteins are provided.

ORFs 13 and 14 encode partitioning proteins ParB and ParA respectively, involved in plasmid segregation during cell division. A putative toxin/antitoxin system (PemK-like and RelB-like proteins) contributing to plasmid stability is encoded by ORFs 16 and 17. pOENI-1 does not encode the full set of proteins required for plasmid conjugation, but only two (ORFs 1 and 3). ORFs 7 and 8 code for a TetR transcriptional regulator and a putative permease of the TauE family. Permeases of this family are known to act as sulfite transporters [47], [48]. It is likely that ORF 7 and ORF 8 encode proteins that are functionally related since pairs of similar genes were detected in *Lactobacillus mali* (Table 3) and in the *O. oeni* phages fOg30, fOgPSU1 and fOg44 [49]. ORFs 10 and 11 encode a LysR transcriptional regulator and a NADH:flavin oxydoreductase of the old yellow enzyme (OYE) family, group 4. The biological role of OYEs is still poorly understood, but they can contribute to the oxidative or general stress response [50], [51], [52]. The rest of pOENI-1 includes a resolvase (ORF 9), a transposase (ORF 12) and five hypothetical proteins of 69 to 128 amino acids in length.

Investigations in public databases revealed that several ORFs of pOENI-1 involved in plasmid maintenance or replication are conserved in other LAB plasmids. The most similar are plasmids pH10 of *Lactobacillus helveticus* H10 (ORFs 1 to 6, ORF 9, ORF 15 and ORFs 18 to 20) and p1 of *Lactobacillus casei* ATCC 334 (ORFs 12 to 20) (Fig. 1B). The TauE and OYE-encoding genes of pOENI-1 were not detected in these plasmids or in other plasmids previously described. Blast analyses revealed that two of the three *O. oeni* genomes available in databases contain sequences related to pOENI-1. The almost complete plasmid sequence is disseminated in four contigs in the draft genome of *O. oeni* AWRIB429, an industrial starter named C10. Only a 104-bp fragment of ORF 15 (*repA*) was not detected in this draft genome. In addition, O. oeni ATCC BAA 1163 – a strain that is known for its poor fermentation capacities in wine [53] – contains chromosomal genes sharing above 98% identity to pOENI-1 ORFs 9 to 12 (Figure 1C). The four genes of *O. oeni* ATCC BAA 1163 are contiguous and form a cluster along with six additional genes unrelated to pOENI-1. This cluster is inserted into the gene OEOE_0812 and bordered by an 8-bp repeated sequence.

### Distribution of pOENI-1, pOENI-1v2 and plasmid genes amongst *O. oeni* strains

**Figure 2:**
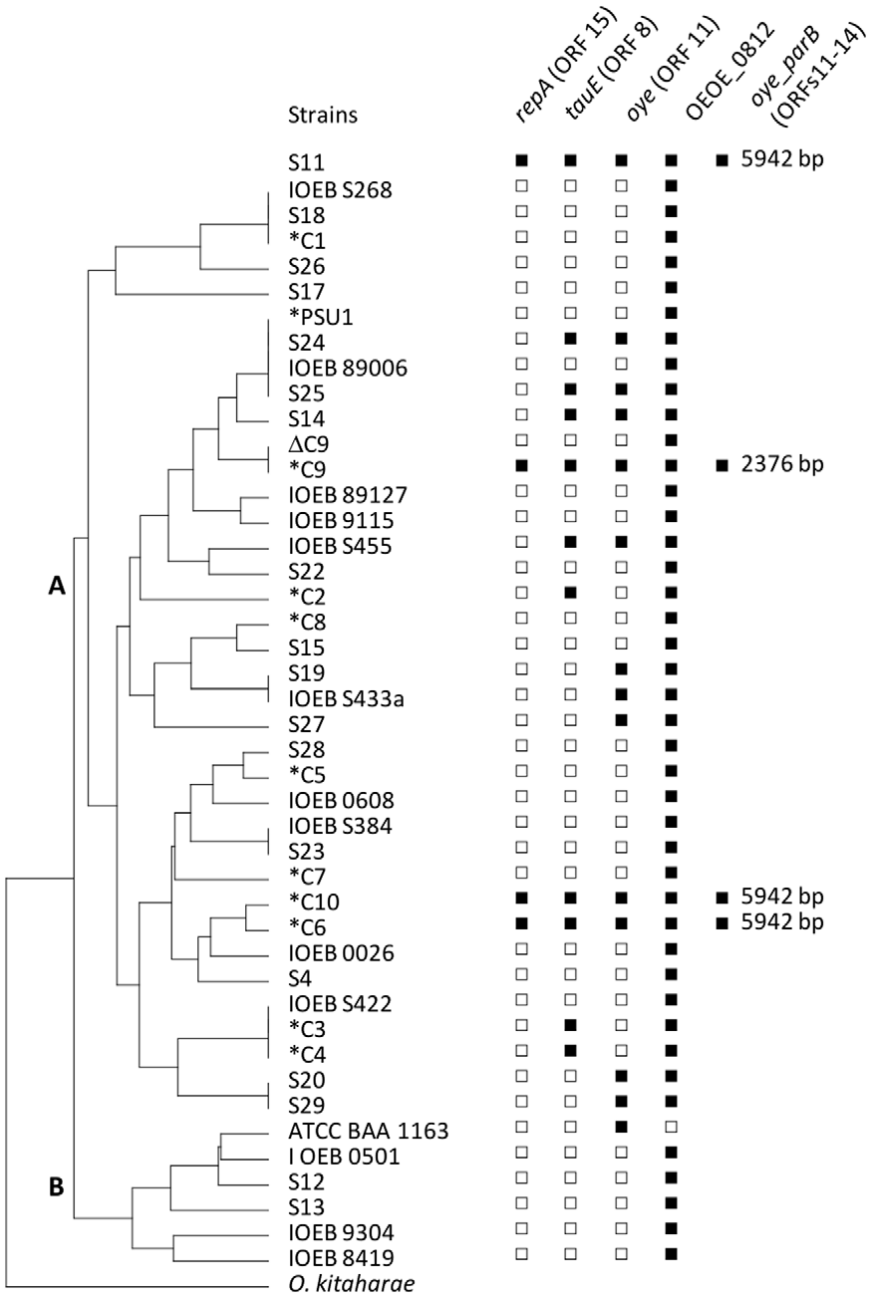
Distribution of pOENI-1 genes in 44 *O. oeni* strains. The dendrogram was constructed from DNA banding patterns obtained by NotI-PFGE analysis of 44 *O. oeni* strains. *Oenococcus kitaharae* was used as outgroup. Strain S11 was positioned on the basis of MLST data since no NotI-PFGE pattern was obtained for this strain. The presence (filled square) or absence (empty squares) of plasmid genes *repA*, *tauE*, *oye* and of the chromosomal gene OEOE_0812 were determined by PCR. The presence/absence of a region encompassing the *oye* and *parB* genes was also investigated. IOEB: Institute of oenology of Bordeaux, S: SARCO, ATCC: American type culture collection. Indutrial strains are marked with asterisks. Letters A and B in the dendrogram represent two phylogenetic groups of strains [36].

Forty-four *O. oeni* strains from diverse origins were analyzed to examine the frequency of pOENI-1 in the species: 11 industrial starters from seven companies, 15 laboratory strains collected between 1983 and 2009, and 18 strains isolated from red and white wines during this study. The phylogenetic relationships of the strains were determined by REA-PFGE and MLST analyses. The dendrogram depicted in Figure 2 shows that all strains belong to two major phylogenetic lineages as suggested in previous studies [36], [54]. The presence of pOENI-1 was investigated by a PCR-based strategy targeting the plasmid ORFs 15 (*repA*), 8 (*tauE*) and 11 (*oye*). The three ORFs were detected only in four strains, including three industrial starters (C9, C10, C6) and a new isolate (S11) (Figure 2). A second series of three PCRs targeting large regions of pOENI-1 confirmed that these strains contain complete plasmids (see primers list in Table 2). However, the region extending from ORFs 11 to 13 was 2376-bp long in pOENI-1 of strain C9, whereas it extended over 5942-bp in the other strains (Figure 2). The sequence of this larger fragment was determined by analyzing the genome of *O. oeni* S11 by the 454 technology. Genome sequence analysis revealed that *O. oeni* S11 holds a 21,926-bp plasmid that was named pOENI-1v2. It carries the same 20 ORFs as pOENI-1 and six additional ORFs located between ORFs 12 and 13, which accounts for the larger PCR products described above. These ORFs encode a recombinase, transposases and hypothetical proteins (Figure 1D). All other parts of pOENI-1 and pOENI1-v2 are very similar (>99% sequence identity), except that two mutations disrupting ORFs 4 and 20 in pOENI-1 are not detected in pOENI-1v2 in which these ORFs encode full-length proteins. It is possible that strains C10 and C6 have acquired pOENI-1v2 by vertical inheritance as they occupy close positions in the dendrogram depicted in Figure 2. In contrast, strains C9 and S11 are distantly related, suggesting that plasmids were also transmitted via horizontal transfer events.

PCR screening did not disclose any other strain containing a full plasmid sequence since *repA* was present only in the four strains above-mentioned. However, *tauE* and *oye* were detected together or separately in 7 and 10 additional strains, respectively (Figure 2). Of the 11 industrial starters analyzed in this work, six contained *tauE* (C9, C10, C6, C4, C3 and C2), while the gene *oye* was found only in the three starters carrying a plasmid. The *tauE* and *oye* genes are randomly distributed among strains, suggesting that they were mostly acquired through horizontal gene transfer events.

The number of plasmids per cell was determined by qPCR analysis of a plasmid gene (*oye*) and a chromosomal gene (*rpoB*). qPCR tests and standard curves were developed for both genes and used to determine the ratios *oye*/*rpoB* in *O. oeni* strains carrying pOENI-1, pOENI-1v2 or the *oye* gene alone (Table 4). pOENI-1 and pOENI-1v2 were present at 3.3 to 4.7 copies per cell, respectively. Bacteria carrying *oye* but no plasmid have approximately one copy of *oye* gene per cell, which is consistent with a chromosomal localization. A complete gene OEOE_0812 was detected in these strains, indicating that their copy of *oye* was not inserted in the same position as in strain ATCC BAA 1163 (Fig. 1C).

**Table 4:** Plasmid/*oye* copy number.

Strain	Plasmid type	*oye* (copies.µl^−1^)	*rpoB* (copies.µl^−1^)	ratio *oye*/*rpoB*
**C9**	pOENI-1	7.7×10^3^±2.8×10^3^	2.1×10^3^±0.7×10^3^	3.7±0.6
**C10**	pOENI-1v2	6.6×10^4^±4.9×10^4^	1.4×10^4^±1.0×10^4^	4.7±0.2
**C6**	pOENI-1v2	2.3×10^4^±0.5×10^4^	6.7×10^3^±2.4×10^3^	3.7±1.2
**S11**	pOENI-1v2	6.8×10^4^±1.8×10^4^	2.1×10^3^±0.4×10^3^	3.3±0.4
**Type S14** a	no plasmid	-	-	1.1±0.2

a average plasmid copy number in stains carrying a pOENI-1 like plasmid: S14, S19, S20, S24, S25, S27, S29, IOEB S433a, IOEB S455.

### Comparison of plasmid-containing and plasmid-free cells during wine fermentations

**Figure 3:**
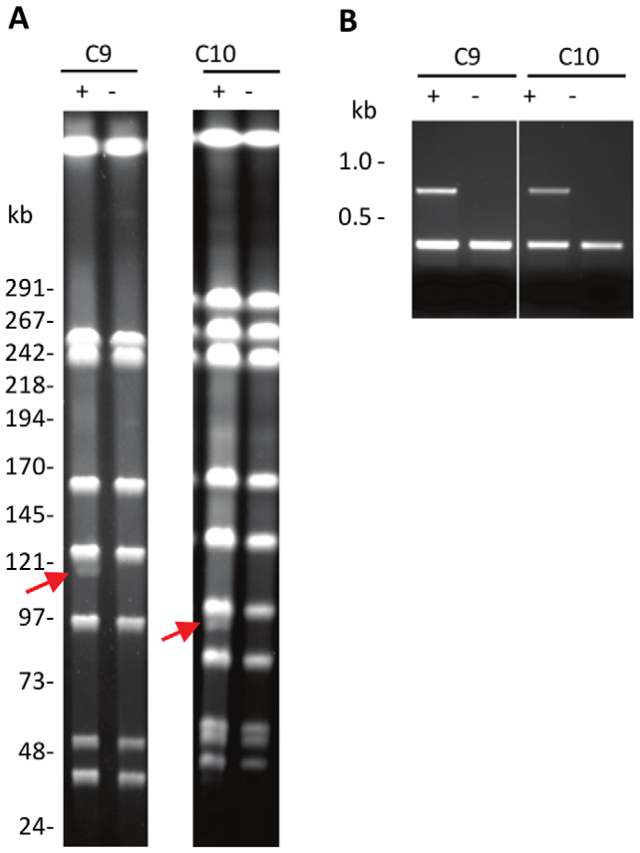
Control of plasmid-free strains. A. Comparison of NotI-PFGE patterns of plasmid containing strains (C9+, C10+) and isogenic plasmid-less derivatives (C9− and C10−). Red arrows indicate bands corresponding to plasmids in strains C9+ and C10+. B. Absence of plasmids in strains C9− and C10− was confirmed by multiplex PCR targeting a plasmid gene (ORF 20, 821-bp PCR product) and a chromosomal gene (*mleA*, 430-bp PCR product).

Detection of plasmids pOENI-1 and pOENI-1v2 in three industrial starters prompted us to examine whether they contribute to the technological properties of their hosts. In order to compare strains sharing the same genetic background, we have generated plasmid-less derivatives of strains C9 and C10 by growing cells in liquid GJ medium during approximately 20 generations prior to plate samples and to test colonies using a plasmid-specific PCR test. Analysis of 68 and 30 colonies of *O. oeni* C9 and C10 allowed for the identification of two and one plasmid-less mutants, respectively. Controls performed by REA-PFGE and PCR assays confirmed that the mutants share the same genetic background as parental strains and have lost the plasmids (Figure 3).

**Figure 4:**
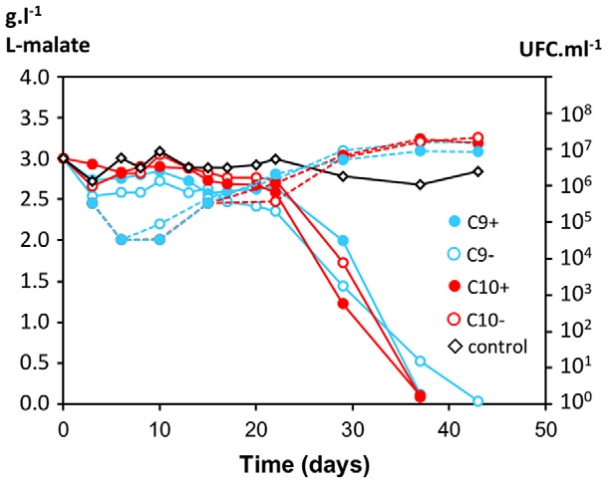
Comparison of MLF kinetics of isogenic strains with/without plasmids. Kinetics of L-malate conversion (solid lines) and monitoring of cell population (dotted lines) were monitored following inoculation of bacteria to 10^7^ cells ml^−1^ in a red wine containing 3 g l^−1^ L-malate. A control was performed without added bacteria. Values are means of two biological replicates.

To determine if plasmids confer an advantage during MLF, strains with (C9+, C10+) and without (C9−, C10−) plasmids were produced under industrial conditions, freeze-dried and tested in micro-vinification assays. They were inoculated to 10^7^.ml^−1^ in a red wine and consumption of L-malate and bacterial populations were monitored until completion of MLF. Bacterial populations evolved similarly whichever the strain. They declined rapidly after inoculation in wine, started to grow after 5 to 10 days and showed similar growth curves during all the rest of the experiments. Bacteria started to consume significantly L-malate after a lag phase of about 20 days and completed MLF in 37 to 43 days following inoculation (Figure 4). Only for strains C9+ and C9− a slight difference was noticed, strain C9+ being able to achieve MLF six days before strain C9−. This difference was also observed and more pronounced (>10-days difference) when this assay was performed in other wines (data not shown). In contrast, strains C10+ and C10− showed similar kinetics of MLF in all trials.

**Figure 5:**
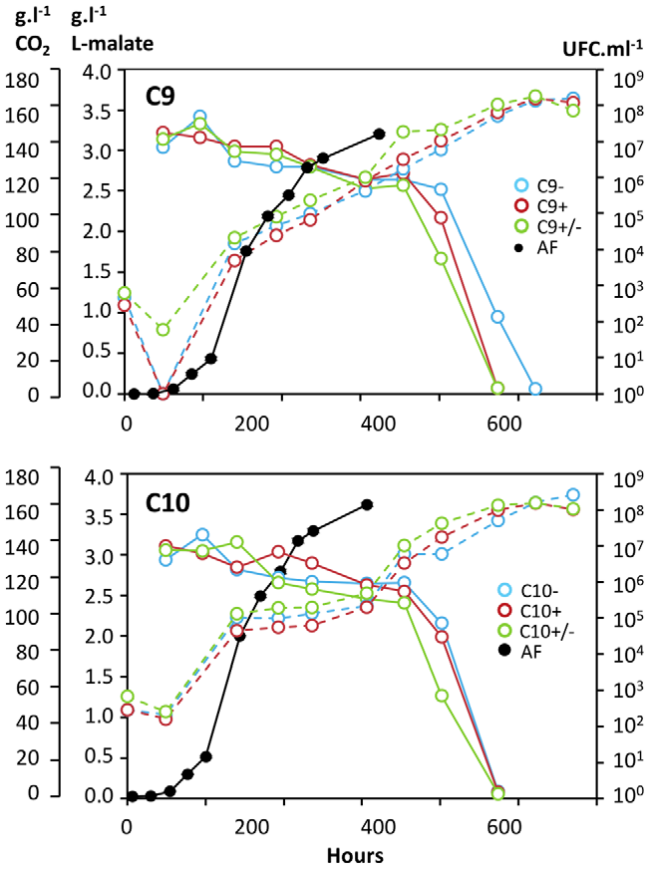
Comparison of growth in wine of isogenic strains with/without plasmids. Kinetics of alcoholic fermentation (CO_2_ released, dark line), MLF (colored solid lines) and bacterial populations (colored dotted lines) were monitored in a sterile grape must inoculated with industrial wine yeasts and 10^3^.ml^−1^ bacteria carrying pOENI-1 or pOENI-1v2 (red lines), bacteria without plasmids (blue lines) or a mixture of both (green lines). Kinetics of AF (dark symbols) is the mean of the three experiments.

A second series of tests was performed to determine if plasmids confer a growth advantage during the phases that precede MLF. Bacteria were inoculated in a sterile grape must to 10^3^ cells.ml^−1^ at the same time as yeasts to perform alcoholic fermentation (Figure 5). The growth of strains with or without plasmids was similar during AF and the ensuing MLF. Additional trials consisting in mixtures of C9+/C9− or C10+/C10− cells inoculated as above showed the same growth curves. The kinetics of MLF were also very similar in all cases, except that strain C9+ achieved MLF two days prior to C9−. To determine if the plasmids were stable during cell growth in wine, samples were collected at the inoculation time, at the end of AF and at the end of MLF and they were plated to isolate colonies that were tested in PCR assays specific for the plasmids (Table 5). At inoculation, C9+ and C10+ contained only 90% of plasmid-containing cells, which denotes the instability of the plasmids during precultures in laboratory. During AF and MLF (approx. 20 generations), plasmids were stable since they were detected in 90 to 100% of the cells. In samples inoculated with equal amounts of plasmid-carrying and plasmid-free cells, the C9+/C9− ratio increased from 48/52 at inoculation time to 57/43 at the end of MLF. However an opposite tendency was noticed for the mixture C10+/C10− (Table 5). We concluded that plasmids did not confer a clear advantage to their host cells in laboratory trials, although they were stably maintained during growth in wine.

**Table 5:** Percentage of plasmid-carrying/plasmid-free cells at different times of winemaking.

Experiment^a^	Inoculation	End of AF	End of MLF
**C9−**	100	100	100
**C9+**	90	70	90
**C9+/C9−**	42/58	46/54	57/43
**C10−**	100	100	100
**C10+**	90	90	100
**C10+/C10−**	50/50	49/51	45/55

a strains inoculated in must.

### Detection of plasmids and plasmid genes in wines

**Figure 6:**
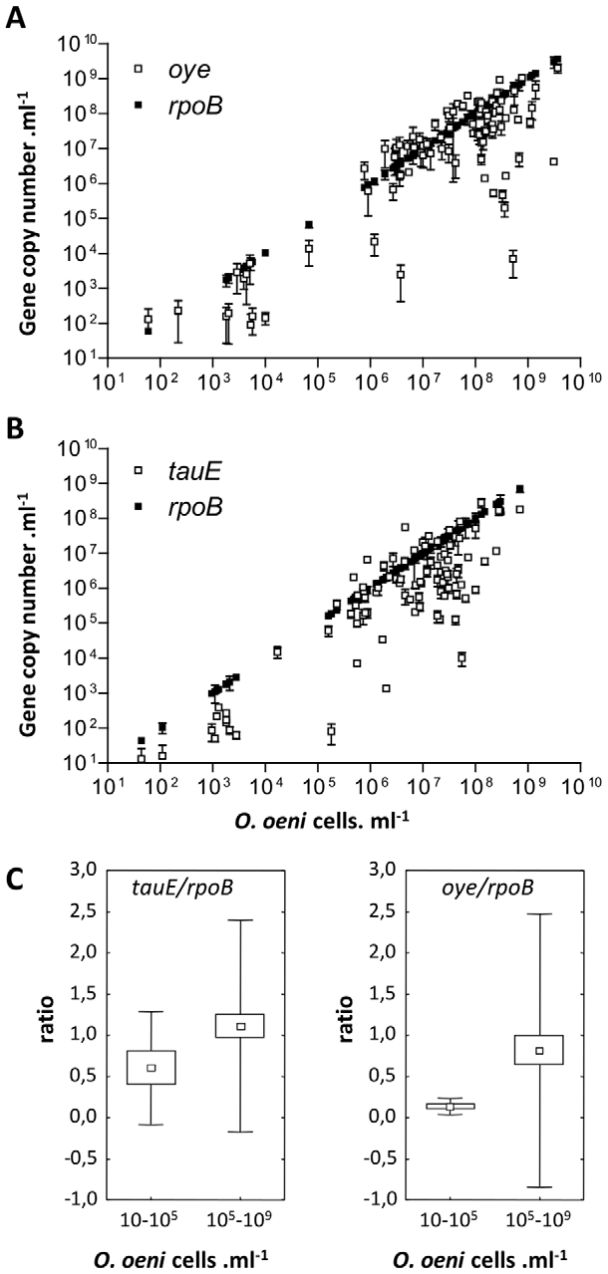
Frequency of *tauE* and *oye* genes during wine fermentations. A. B. The *oye* and *tauE* gene were quantified by qPCR analysis of 95 samples of must or wine collected at different stages of winemaking. Data obtained from *rpoB* quantifications were plotted on the x-axis to appraise the *O. oeni* population and on the y-axis to make easier the comparison between the *O. oeni* population (*rpoB*, filled squares) and the *tauE* or *oye* copy number (empty squares). Data are means of two independent determinations. C. The average ratios of *tauE/rpoB* or *oye/rpoB* were calculated from samples collected in must or AF (10–10^5^ cells.ml^−1^) and during MLF (10^5^–10^9^ cells.ml^−1^). The boxes and lines represent the means (small squares), standard errors (large squares) and standard deviations (lines).

To determine if the plasmids have a technological significance during real winemaking, we have investigated their presence in bacteria of 95 samples collected in 86 wineries at different phases of wine fermentations (must, AF, MLF). Microbial DNAs were purified from each sample and used as template in quantitative PCR assays to determine the *tauE* and *oye* copy numbers. The chromosomal gene *rpoB* was also quantified in order to assess the total *O. oeni* population. As anticipated, the *O. oeni* population ranged from 10 to 10^5^ cells.ml^−1^ in samples collected in must and AF, while it reached up to 10^9^ cells.ml^-1^ during MLF (Figure 6). The genes *tauE* and *oye* were detected in all samples. They were present at high copy numbers in the vast majority of samples collected during MLF: above 10^6^ copies.ml^−1^ in 55.8% (*tauE*) and 78.9% (*oye*) of samples. The average ratios of *tauE/rpoB* and *oye/rpoB* were calculated in samples collected before and during MLF (Figure 6C). The ratios were below 0.6 in must/AF samples and above 0.9 during MLF. This suggests that bacteria carrying *tauE* and *oye* were underrepresented before MLF but proliferated during AF and became predominant in MLF.

**Figure 7:**
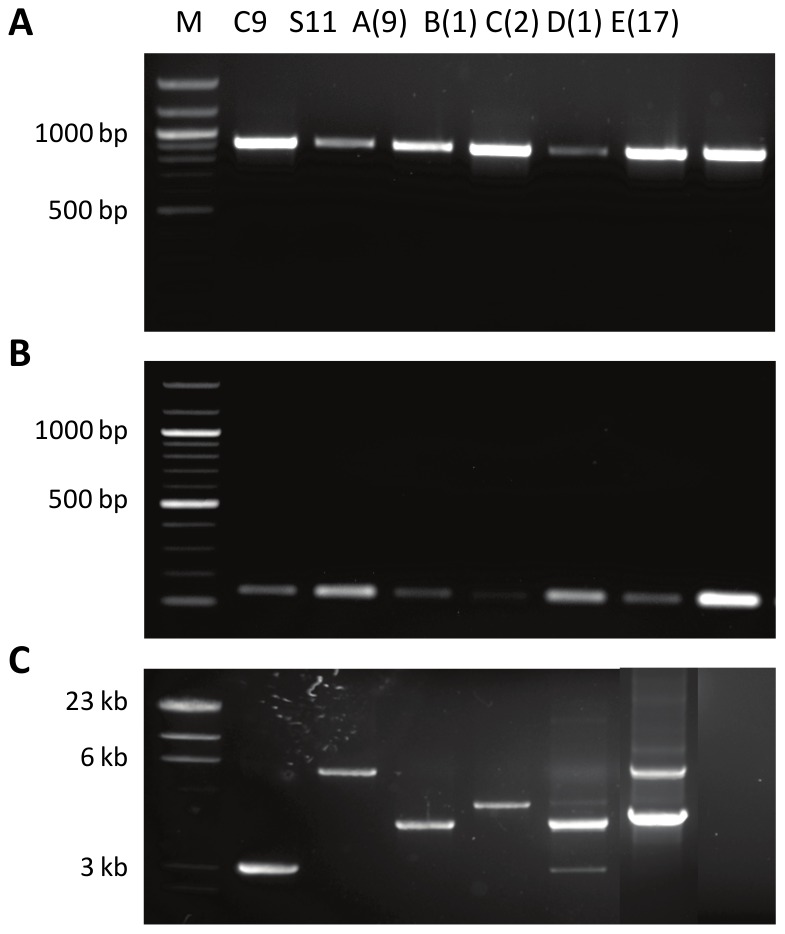
PCR detection of pOENI-1 and related plasmids in wines. PCR assays were performed using DNA templates from *O. oeni* C9 (pOENI-1), *O. oeni* S11 (pOENI-1v2) and 30 samples of wine collected during MLF (A–E). The number of samples sharing the same PCR product is indicated in parentheses. The primers allowed detection of pOENI-1 *repA* (panel A), *tauE* (panel B) and a region extending from ORF11 (*oye*) to ORF 13 (*parB*) (panel C). M: DNA size markers.

Thirty samples of wines were further tested by PCR to determine if the genes *tauE* and *oye* were located on plasmids resembling pOENI-1 or pOENI-1v2. PCR assays were performed using primers bordering a plasmid region that extends from ORF 11 to ORF 13 and extends over 2376-bp in pOENI-1 and 5942-bp in pOENI-1v2. PCR products were obtained for 13 samples (Figure 7). Three samples contained a mixture of products of two different sizes, which denote that cells carrying different plasmids were present together in some samples. Only two and one samples had PCR products of molecular sizes expected for pOENI-1 and pOENI-1-v2, respectively. The other PCR products had intermediate sizes of around 4.0 kb, suggesting that additional forms of pOENI-1 can be encountered. The 17 samples that did not generate PCR products were further analyzed in PCR assays targeting specifically *repA, oye* or *tauE*. They all contain a copy of *repA, oye* and *tauE*, but we could not assess whether the genes were located on plasmids unrelated to pOENI-1 or on the chromosome (not shown).

## Discussion

### Plasmids of the “pOENI-1 family”

pOENI-1 and pOENI-1v2 are the first large plasmids described in *O. oeni*. The presence of large plasmids in this species was known from previous works [31]–[35], but no sequence was available. However, during the preparation of this manuscript, Borneman and coworkers have reported the sequences of 11 *O. oeni* genomes and found plasmids in four of them (discussed below) [55]. The plasmids pOENI-1 and pOENI-1v2 carry a majority of ORFs involved in maintenance and replication but also a few ones encoding proteins that can benefit to their host cells, such as TauE and Oye. They share a limited sequence similarity with plasmids found in other LAB. The most similar is plasmid p1 of *L. casei* [56] that shares similarities over a 6-kb region comprising the origin of replication and proteins ParA/ParB (partitioning), RepA (replication), RelB/PemK (toxin/antitoxin system of maintenance) and TraA (transfer). pOENI-1 and pOENI-1v2 most likely use a theta-mode of replication since they encode a RepA protein that is conserved in theta-type plasmids described in other LAB [44]. Their origin of replication also is typical of such plasmids [45], [46]. It is noteworthy that they were detected at a low copy number (3 to 5 copies per cell), which is consistent with plasmids using this mode of replication. There is no doubt that pOENI-1 and pOENI-1v2 derive from each other since they share extensive sequence identity (>99% nucleotide sequence identity over the whole pOENI-1 sequence). Their main difference is a 3.5-kb insert that is present between ORFs 12 and 13 in pOENI1-v2 and absent in pOENI-1. This insert encodes recombinase, transposases and hypothetical proteins without apparent functional role. The plasmids also differ at several nucleotide positions. pOENI-1v2 contains full-length ORFs 4 and 20 coding for a glycerate dehydrogenase and a DNA nickase, respectively, whereas these ORFs are interrupted by early stop codons in pOENI-1. Therefore, we suppose that pOENI-1v2 has preceded pOENI-1 during evolution. However, it is difficult to assess whether pOENI-1v2 is the direct progenitor because analysis of wine samples suggest that there are at least two additional plasmids in this “pOENI-1 family”. They differ from the former in the insert region which has intermediate sizes between pOENI-1 and pOENI-1v2 (see Figure 7). It seems that one of these intermediate forms is more frequent in wines than pOENI-1 or pOENI-1v2. It was detected in 12 samples of wines collected during MLF, from a total of 13 samples containing the plasmids.

pOENI-1 and pOENI-1v2 are not conjugative because they do not carry the full set of proteins required for conjugation. However, pOENI-1v2 encodes a nickase (ORF 20, disrupted in pOENI-1) that is typical of mobilizable LAB plasmids [44]. This suggests that at least pOENI-1v2 can propagate by mobilization. In fact, the distribution of plasmids in 44 *O. oeni* strains analyzed in this work supports well the hypothesis that they were horizontally exchanged. Of the four strains that carry a copy of pOENI-1 (C9) or pOENI-1v2 (C10, C6, S11), only two are closely related in dendrograms and phylogenetic trees constructed from PFGE or MLST analyses (C10, C6). The third strain carrying pOENI-1v2 (S11) and strain C9 with pOENI-1 are positioned on distant branches. This distribution most likely results from a dissemination of plasmids via horizontal transfer events.

### Potential role of plasmids

Previous studies have demonstrated the importance of plasmids in conferring valuable properties to industrial LAB strains [24], [43]. Plasmids of the pOENI-1 family encode the proteins TauE and OYE, which could be useful for wine bacteria. TauE belongs to a family of membrane transporters involved in the import/export of sulfites or sulfur-containing compounds. Enzymes of this family were characterized as exporters in *Cupriavidus necator* and *Neptuniibacter caesariensis*, in which they contribute to the metabolism of taurine (2-aminoethanesulfonate) [48], [57]. Sulfites can be added at different phases of winemaking for their antioxidant and antimicrobial properties. They are also naturally produced by yeasts during alcoholic fermentation. High concentrations of sulfites in wine may prevent the development of bacteria and avoid MLF to occur [58]. The protein OYE could be also advantageous for wine bacteria. It has the functional domains conserved in NADH:flavin oxidoreductases of the large “old yellow enzymes” family. These enzymes are involved in diverse biological functions including stress response in a number of living cells [52], [59]–[64]. They were not characterized in LAB so far, but identified in *Bacillus subtilis* in which they are expressed in response to oxidative stress and acidification of the cytosol [50], [65]. However, our comparison of strains carrying or not the plasmids has not revealed clear phenotypic differences during MLF or during growth in wine. Strain C9 carrying pOENI-1 has repeatedly completed MLF a few days before its plasmid-less derivative, but this was not confirmed by comparing strains C10 carrying or not pOENI-1v2. Therefore it is yet unclear whether the plasmids confer a significant advantage during growth in wine and what could be this advantage. Further analyses of plasmid genes expression should help to solve this issue. It is noteworthy that besides TauE and OYE, the plasmids encode several hypothetical proteins which could be important for bacteria.

### Predominance of plasmids in starter strains and indigenous strains performing MLF

Despite the absence of clear evidence for a role of the plasmids, their distribution among *O. oeni* strains and bacteria present in wine suggests that they could contribute to the fitness of bacteria performing MLF. Of the 44 strains of our study collection, which included 11 industrial starters and 33 non-starter strains, the plasmids were present in only four strains: three starters (C9, C10, C6) and a new isolate (S11) that performs well MLF (data not shown). This represents a frequency of 27% in starters (3/11) and 3% in other strains (1/33). In their recent study, Borneman and coworkers have also detected plasmids in the genomes of four of the 11 strains that they have analyzed: two starters (AWRIB419, AWRIB422) and two strains (AWRIB565, AWRIB576) sharing close genetic relationships with the starter AWRIB429 [55]. By examining these new genomes, we have found that they contain pOENI-1 (AWRIB419), pOENI-1v2 (AWRIB565, AWRIB576) and a divergent pOENI-1-like plasmid containing a different RepA protein and apparently lacking the partitioning proteins (AWRIB422). In addition, although this was not mentioned by the authors, the starter AWRIB429 also contains a plasmid. This starter was investigated in our study under the name “C10” and our results prove that it contains the plasmid pOENI-1v2 (Figures 2 and 3). This plasmid sequence is split into four contigs of the published sequence of AWRIB429 (ACSE00000000). These new findings confirm the high frequency of plasmids of the pOENI-1 family in starter strains. They are detected to date in eight strains, among which there are five starters (C6, C9, C10 = AWRIB429, AWRIB419, AWRIB422), two strains closely related to one of these starters (AWRIB565, AWRIB576) and one strain performing well MLF (S11). It is noteworthy that we have also detected a large fragment of the plasmid which includes the ORF encoding TauE in the genome sequence of another starter (AWRIB548, named C4 in our study). In this strain, a fragment of the plasmid has possibly been integrated in the chromosome (sequence acc. number ALAH00000000). The same situation was detected in the genome sequence of starter C3 (unpublished data) that is genetically closely related to C4 (Figure 2).

Our results showed also that the plasmids or the plasmid-encoded genes are frequent in indigenous bacteria performing MLF. The genes *tauE* and *oye* were detected in all of the 95 samples of wine analyzed in this work. They were particularly abundant in samples collected during MLF, reaching the same level as the total cell population. It is very unlikely that the plasmid-encoded genes confer the capacity to survive in wine and to perform MLF. Wine is a complex and harsh environment for most microorganisms [1]. The ability of bacteria to survive in wine and to conduct efficiently MLF involves many genes, many of which have already been described [11]–[18]. However, the predominance of pOENI-1-like plasmids and plasmid-encoded genes in starter strains and indigenous bacteria performing spontaneous MLF indicates that they contribute positively to the fitness of these bacteria during winemaking.
